# The role of lncRNAs and circRNAs in the PD-1/PD-L1 pathway in cancer immunotherapy

**DOI:** 10.1186/s12943-021-01406-7

**Published:** 2021-09-08

**Authors:** Wenxiao Jiang, Shuya Pan, Xin Chen, Zhi-wei Wang, Xueqiong Zhu

**Affiliations:** Departmant of Obstetrics and Gynecology, The Second Affiliated Hospital of Wenzhou Medical University, No. 109 Xueyuan Xi Road, Wenzhou, 325027 Zhejiang China

**Keywords:** LncRNAs, Cancer, CircRNAs, PD-1, PD-L1, Immunotherapy

## Abstract

Cancer immunotherapy has recently shown promising antitumor effects in various types of tumors. Among all immune checkpoints, the PD-1/PD-L1 pathway plays an important role in the immune evasion of tumor cells, making it a potent target in antitumor immunity. Accordingly, antibodies targeting the PD-1/PD-L1 pathway have been developed to attack tumor cells; however, resistance to immune therapy remains to be solved. Hence, identification of the underlying modulators of the PD-1/PD-L1 pathway is of significant importance to understand the mechanisms of antitumor immunotherapy. Long noncoding RNAs (lncRNAs) and circular RNAs (circRNAs) have been identified to regulate the PD-1/PD-L1 pathway, leading to participation in the immune response and immunotherapy. Therefore, this review focuses on the functions of lncRNAs and circRNAs in regulation of the PD-1/PD-L1 axis in tumorigenesis and tumor progression. We hope this review will stimulate research to supply more precise and effective cancer immune checkpoint therapies for a large number of tumors.

## Introduction

The human immune system plays an important role in maintenance of homeostasis by discriminating and eliminating aberrant cells, including pathogens and cancer cells, and in this way, protecting the human body from cells of endogenous and exogenous origins [1]. Accordingly, cancer immunotherapy, which stimulates the immune system to create promising antitumor effects, has been developed to attack tumors [2]. The discovery of CTLA-4 [3] and PD-1 [4], which were identified as immune checkpoints acting as a brake in immune function, was awarded the 2018 Nobel Prize in Physiology or Medicine. With connection of the T cell receptor on the T cell surface and peptide-MHC on target cells, immune checkpoints are inhibited, consequently leading to dysfunction of T cells and antitumor results [5].

Among all immune checkpoints, the PD-1/PD-L1 signaling pathway has received increasing attention due to its proven outstanding effectiveness as an immune therapeutic target in a wide range of tumors, including bladder cancer, lung cancer, and pancreatic cancer [6–8]. However, the response to anti-PD-1/PD-L1 therapy differs among different malignancies and patients, and multiple mechanisms have been found to hinder tumor immunity during tumorigenesis [9–11]. This finding urges current researchers to evaluate the underlying mechanisms by which the PD-1/PD-L1 pathway is regulated in tumorigenesis. Recently, noncoding RNAs have been reported to participate in regulation of PD-1/PD-L1 pathway in carcinogenesis [12–15]. Hence, in this review, we describe the regulation of the PD-1/PD-L1 pathway by lncRNAs and circRNAs in tumors and discuss how these regulatory networks modulate the PD-1/PD-L1 pathway in cancer immunotherapy. Regulation of lncRNAs and circRNAs can be used to design more precise and effective immune therapies by targeting immune checkpoints.

### PD-1/PD-L1 pathway

**Fig. 1:**
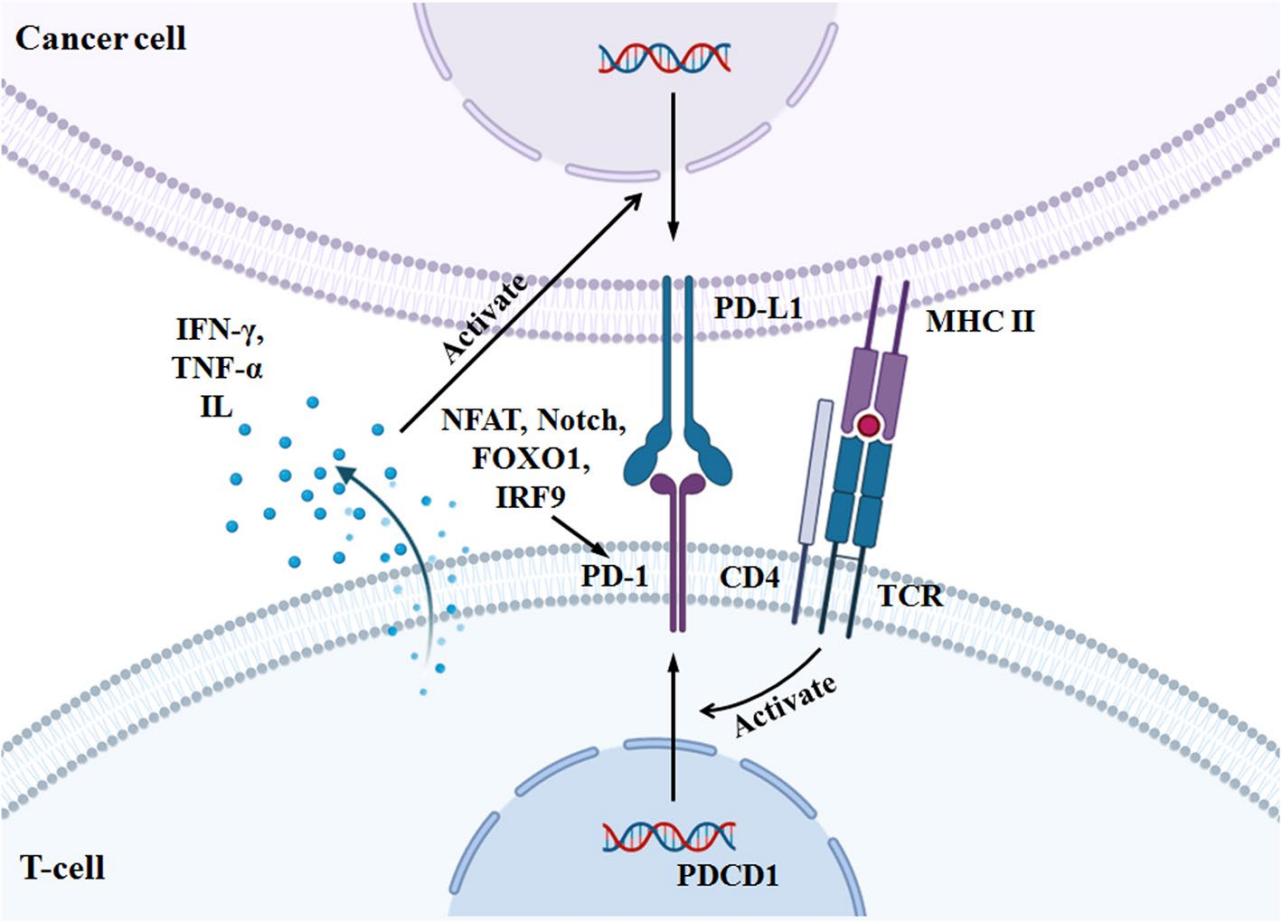
Several factors regulate the PD-1/PD-L1 pathway

PD-1/PD-L1 is an important component of tumor immunosuppression and has been considered a critical issue in immune therapeutic research [16]. PD-1, also known as cluster of differentiation 279 (CD279), was first discovered in 2B4-11 (murine T-cell hybridoma) cells and interleukin-3 (IL-3)-deprived LyD9 cells in 1992 by Tasuku Honjo [4], and then in 1999, its cognate ligand B7 homolog 1 (B7-H1, later termed as PD-L1) was identified [17]. PD-1 is a 55 kDa type I transmembrane glycoprotein and has 288 amino acids with three distinct domains: an extracellular N-terminal domain, a transmembrane domain and an intracellular tail at the N and C ends [18]. PD-1 has 15% similarity to CD28, 20% similarity to CTLA4, and 13% similarity to induced T-cell costimulators [19]. As a major immune checkpoint receptor, PD-1 belongs to the immunoglobulin gene superfamily and is expressed on different immune cells, including T cells, B cells, NK cells, macrophages, DCs, and monocytes [16, 20, 21]. Importantly, PD-1 functions as a coinhibitory receptor that promotes T-cell activation, cytokine production and cytotoxicity by binding to its ligands [16]. In this way, antigen-presenting cells (APCs) can take up antigen released from tumor cells and present it to T cells. Tumor cells can also present antigens to activate T cells in the context of major histocompatibility complex (MHC). Upon T cell activation, PD-1 can downregulate immune responses through involvement of PD-L1 on tumor cells. Prolonged T cell receptor (TCR) stimulation can upregulate PD-1 expression. The PD-1/PD-L1 interaction inhibits T cell proliferation and interferon-γ (IFN-γ) production, resulting in a reduction in T cell survival [22]. On the other hand, a variety of transcription factors are capable of triggering PD-1 expression, such as NFAT, NOTCH, FOXO1 and interferon (IFN) regulatory factor 9 (IRF9) [23] (Fig. 1). Conserved regions B and C (CR-B and CR-C) are the upstream regulatory regions of the PD-1 gene (PDCD1) and are critical for PD-1 expression. The CR-C region binds to NFATc1 (NFAT2) in CD4 + and CD8 + cells through its binding site to NFAT, and the CR-B region can be attached via c-FOS when detecting antigen (Ag) in naive T cells after stimulation with T-cell receptor (TCR), resulting in induction of PD-1 expression [24]. Similarly, other transcription factors (including IFN-α, IRF9, FOXO1) and cytokines (such as IL-10 and IL-27) can induce PD-1 expression by binding to the PD-1 promoter [25, 26]. PD-1 has been referred to as a critical inhibitor in adaptive and innate immune systems and stands out in immune therapy forB-cell lymphomas [27].

As one PD-1 ligand, PD-L1 (also called CD274) belongs to the B7 series and is a 33 KDa type 1 transmembrane glycoprotein. PD-L1 has 290 amino acids and two protein domains, the IgV and IgC domains, in its extracellular region [28]. PD-L1 expression is commonly detected on the membranes of the surrounding cells in the tumor microenvironment, including T cells, B cells, APCs, DCs and monocytes, as well as different types of cancer cells [29–31]. Notably, the PD-L1 expression level in tumor cells can be modulated at multiple levels, such as genetic aberrations and epigenetic regulation via extrinsic and intrinsic oncogenic signaling pathways [32]. Epigenetic modulation includes miRNAs, histone modification, DNA methylation, and PTM. For example, proinflammatory cytokines, such as IFN-γ and TNF-α, secreted by tumors, induce upregulation of PD-L1 on the tumor cell surface, which is considered an “adaptive immune mechanism” to escape antitumor responses [31, 33]. The antitumor immune activity of PD-L1 is dependent on its binding to PD-1. The PD-1/PD-L1 pathway primarily functions in blockade of TCR and costimulatory signals. In detail, the binding between PD-L1 on cancer cells and the PD-1 receptor on immune T cells induces phosphorylation of the tyrosine-based inhibitory motif (ITIM) of PD-L1 located in the cytoplasmic domain and of the tyrosine-based switch motif (ITSM) by Src family kinases, which then activates SHP-2 and SHP-1 to inhibit antigen-driven activation of T cells through the T cell receptor pathway [2, 32, 34]. Additionally, PD-L1 expression on several immune cells, such as T cells, APCs and macrophages, can be regulated by different translational factors to reduce antitumor immunity [35]. These translational factors include cytokines produced by infiltrated immune cells, such as IFN-γ, TNF-α, VEGF, IL-4, and IL-10, as well as TLRs, STAT1, and HIF-1α [36, 37]. Based on their inhibitory capabilities in the activity of T cells, the PD-1/PD-L1 pathway can induce immune evasion of tumor cells and interfere with antitumor immunity. In the tumor microenvironment, after recognizing antigens presented on the tumor surface, T cells rapidly express PD-1 and develop into exhausted T cells. Then, exhausted T cells release cytokines, including IFN-γ, TNF-α and IL, to enhance PD-L1 expression, which consequently inhibits the activation of T cells and reduces the immune response, resulting in tolerance of the immune system to self-antigens [38, 39]. On the other hand, the PD-1/PD-L1 pathway is regulated by a variety of molecular pathways in tumorigenesis, including the PI3K/AKT, MAPK, JAK/STAT, WNT, NF-κB and Hedgehog (Hh) pathways [7], which can function as upstream mediators of the PD-1/PD-L1 axis in different tumor cells. For example, activation of the PI3K/Akt/mTOR signaling pathway is significantly related to the recovery of exhausted CD8 + T cells by PD-1/PD-L1 blockade in gastrointestinal stromal tumors [40]. Moreover, inhibition of the MAPK pathway was found to prevent EGF- and IFN-γ-induced PD-L1 expression in lung adenocarcinoma cells [41]. Accordingly, antibodies suppressing the PD-1/PD-L1 pathway have been developed to destroy tumor cells, and in recent decades, PD-1/PD-L1 checkpoint blockade therapies have been suggested for antitumor immunity in the majority of cancer types. In 2014, the PD-1 monoclonal antibody nivolumab was approved by the US FDA for treatment of melanoma [42, 43]. In the following years, the application of other anti-PD-1/PD-L1 pathway monoclonal antibodies, such as atezolizumab, avelumab, durvalumab, and pembrolizumab, or their combination with other therapies were also approved for treatment of many tumor types [44]. However, the clinical resistance of some cancer patients to PD-1/PD-L1 checkpoint blockade therapies makes treatment tougher. Therefore, identification of underlying modulators of the PD-1/PD-L1 pathway is of significant importance in developing novel antitumor drugs for cancer immunotherapy.

### LncRNAs and circRNAs

With the advanced development of the ENCODE project and whole genome and transcriptome sequencing technologies, a class of genomic DNA called ncRNAs, which are not translated into functional proteins, has been identified [45, 46]. Clearly, ncRNAs are unique RNA transcripts that act as modulators of molecular targets and cellular functions [47] and include lncRNAs, snRNAs, snoRNAs, siRNAs, piRNAs and circRNAs [47–49]. LncRNAs and circRNAs (a category of lncRNAs) are important types of ncRNAs involved in regulation of biological processes and many diseases, including human cancer [50]. LncRNAs take part in regulation of gene expression at the epigenetic, transcriptional, and posttranscriptional levels [51] and play a critical role in diverse cellular processes and molecular signaling pathways [52, 53]. CircRNAs can regulate gene expression in part by acting as miRNA sponges and regulating splicing and transcription [54, 55]. Emerging evidence has revealed the critical roles of lncRNAs and circRNAs in regulation of the immune response in tumorigenesis in part via regulation of the PD-1/PD-L1 pathway.

### LncRNAs in regulation of the PD-1/PD-L1 pathway

Emerging evidence has identified that various immune-related lncRNAs are involved in the TME and have significant associations with immune cell infiltration and the cancer cell response to anti-PD-1 immunotherapy in various tumors [56].

### Diffuse large B cell lymphoma (DLBCL)

DLBCL is a highly aggressive hyperplastic disorder originating from B lymphocytes, and approximatey 40% of DLBCL patients present resistance to clinical treatments [57]. Recent studies have reported that many lncRNAs are involved in the immunoregulation of DLBCL, including SMAD5-AS1, which suppresses DLBCL progression via the Wnt/β-catenin pathway [58]; HOTAIR, which predicts a worse prognosis of DLBCL patients [59]; and SNHG14. SNHG14 is upregulated in DLBCL, and its upregulation is related to induction of cancer cell proliferation, migration, EMT, and especially immune evasion. Notably, SNHG14 acts as a critical sponge of miR-5590-3p to elevate the expression level of ZEB1 (an important transcription factor related to the malignant behaviors of many tumors) [60], thereby resulting in activation of the PD-1/PD-L1 pathway and promotion of DLBCL immune evasion [61]. Similarly, lncRNA MALAT1 induces upregulation of PD-L1 expression to promote DLBCL cell proliferation, migration, EMT, and immune escape and to inactivate CD8 + T cells [62]. Importantly, with a luciferase assay, it was found that the effect of MALAT1 was generated by inhibition of miR-195 [62]. These findings demonstrate that lncRNAs can have a potential effect on antitumor immunity in DLBCL cells and can provide tolerance of DLBCL cells to immune responses.

### Ovarian cancer (OC)

OC is the most lethal gynecological malignancy, with an estimated 2.5–5% incidence and associated deaths worldwide [63]. Due to the lack of detectable symptoms in the early stage, over 70% of OC patients are diagnosed with advanced tumors [64]. An increasing amount of evidence has revealed that many lncRNAs participate in regulation of the PD-1/PD-L1 axis as well as the molecular mechanisms underlying ovarian tumorigenesis and therapeutic resistance. For example, Duan et al. reported an anti-immunity function of EMX2OS in OC. High expression of EMX2OS accelerated the proliferation, invasion and sphere formation of cells and amplified tumor growth in mice [65], and more importantly, EMX2OS was found to sponge and inhibit miR-654 and subsequently activate AKT3, resulting in overexpression of PD-L1, which predicted an unfavorable overall survival of OC [65]. In contrast, ectopic expression of PD-L1 can eliminate the tumor suppressive effect of downregulation of EMX2OS and AKT3 or overexpression of miR-654 in OC cells [65]. This result suggests that EMX2OS can be used as a therapeutic target in PD-1/PD-L1 checkpoint blockade therapy for OC. Meanwhile, lncRNA HOTTIP was reported to have the same role in OC, with higher expression in OC tissues than in normal ovarian tissues [66, 67]. HOTTIP is able to induce PD-L1 expression in neutrophils and immune escape and to inhibit T cell immunity, which is attributed to the interaction of HOTTIP with the TME. By targeting the transcription factor c-Jun, HOTTIP induced the secretion of IL-6, and silencing HOTTIP decreased IL-6 expression [67] LncRNA SNHG12 induced PD-L1 expression to accelerate OC immune escape by increasing the expression of IL-6R through recruitment of NF-κB1 to the IL-6R promoter and promotion of IL-6/miR-21 crosstalk between OC cells and M2 macrophages [68].

### Endometrial cancer (EC)

EC is an important gynecological cancer threatening the health of most women. Through cell functional assays, lncRNA Lnc-OC1 was identified to enhance PD-L1 expression, promote cell viability and inhibit cell apoptosis by sponging miR-34a in EC cells [69]. Another group demonstrated that PD-L1 plays a tumor suppressive role in aggressive EC, including poorly differentiated endometrioid adenocarcinoma and serous adenocarcinoma [70]. Eighty-four percent of normal tissues and 12% of the cancer specimens showed positive PD-L1 expression, and PD-L1 staining via immunohistochemistry in normal tissues was found to be deeper than that in EC tissues. Furthermore, upregulation of PD-L1 led to inhibition of cell proliferation and loss of mesenchymal phenotypes [70]. Similarly, using luciferase reporter assays, lncRNA MEG3 was found to upregulate PD-L1 expression by targeting and inactivating miR-216a, leading to inhibition of cell proliferation and loss of mesenchymal phenotypes in aggressive EC, including poorly differentiated endometrioid adenocarcinoma and serous adenocarcinoma [70]. These findings demonstrate that the MEG3/miR-216a/ PD-L1 pathway is involved in aggressive EC.

### Gastric cancer (GC)

Currently, an increasing number of lncRNAs have been identified to show a significant association with GC immunity, especially PD-1/PD-L1 immunotherapy, such as LINC01871 and AC006033 [71]. By using 94 stomach adenocarcinoma datasets from TCGA, a lncRNA model containing 16 lncRNAs was established to classify microsatellite instability (MSI) status for predicting the response to PD-1/PD-L1 immunotherapy [72]. In addition, many lncRNAs are reported to be upregulated in GC, promote gastric tumorigenesis and regulate the PD-1/PD-L1 pathway to induce immune escape, including SNHG15 [73], lncRNA NUTM2A-AS1 [74] and lncRNA HIF1A-AS2 [75]. Mechanistically, SNHG15 directly targeted miR-141 [73], and HIF1A-AS2 sponged miR-429 to positively regulate PD-L1, leading to immune escape [75]. Additionally, lncRNA NUTM2A-AS1 was found to bind to miR-376a to consequently enhance the expression of TET1 and HIF-1A, leading to induction of gastric tumorigenesis and drug resistance, and TET1 interacts with HIF-1A to increase PD-L1 expression levels to promote tumorigenesis and drug resistance in HGC-27 and SNU-1 cells [74].

### Hepatocellular carcinoma (HCC)

Recently, a large number of lncRNAs have been shown to have a significant positive correlation with the activity of PD-L1 in HCC, including MIR155HG [76] and PCED1B-AS1 [77]. By establishing an immune-related lncRNA signature in HCC, it was found that this immune-related lncRNA signature is associated with ICB immunotherapy-related molecules, including IDO1, PD-L1 and PD-L2, as well as infiltration immune cells, including M0 macrophages, Tregs, CD4 memory T cells, and M1 macrophages, and predicted the survival of patients [78], suggesting a close relationship between lncRNAs and the TME, including PD1/PD-L1 pathways. For example, the lncRNA MIAT was significantly associated with the expression of PD-1, PD-L1 and CTLA4, and participates in the immune escape process of HCC by regulating target genes, such as JAK2, SLC6A6, KCND1, MEIS3 and RIN1. As a consequence, lncRNA MIAT is involved in HCC resistance to drugs, such as sorafenib [79].

Abnormal lncRNAs-mediated PD-1/PD-L1 expression may mediate HCC migration and drug resistance through various molecular pathways. CASC11 has been reported to activate NF-κB and the PI3K/AKT/mTOR pathways to further regulate PD-L1 via EIF4A3-mediated upregulation of E2F1, leading to promotion of HCC cell proliferation, mobility, and glucose metabolism [80]. Consistently, another group reported that PCED1B-AS1 is markedly overexpressed in HCC tissues and interacts with hsa-miR-194-5p to increase the expression of PD-L1 and PD-L2, resulting in HCC immune escape and tumorigenesis [77]. In addition, lncRNAs from exosomes also modulate the PD-1/PD-L1 pathway. By isolating exosomes from HCC cells, it was noted that exosomal PCED1B-AS1 promoted the expression of PD-L1 and PD-L2 in recipient HCC cells and inhibited the activity of recipient T cells and macrophages [77]. Moreover, the pseudogene (a special type of lncRNAs) RP11-424C20.2I regulates the immune escape and tumorigenic processes of LIHC at least in part through IFN-γ-mediated CLTA-4 and PD-L1 pathways by sponging miR-378a-3p to increase UHRF1 expression [81]. UHRF1 has been verified to be significantly correlated with immune cell infiltration. In sorafenib-resistant HCC tissues and cells, knockdown of lncRNA KCNQ1OT1 decreased PD-L1 expression by sponging miR-506, leading to the reversal of sorafenib resistance and immune escape [82]. One study demonstrated that lncRNA XIST regulates PD-L1 by targeting miR-194-5p and miR-155-5p in HCC [83].

### Pancreatic cancer (PC)

PC is one of the most lethal diseases with a dismal five-year survival rate. LncRNA PMSB8-AS1 and LINC00473 were highly expressed in PC tissues and cell lines and contributed to upregulation of PD-L1 and promotion of cell proliferation, invasion, and migration in PC [84, 85]. Remarkably, lncRNA PMSB8-AS1 regulated the transcription factor STAT1 by interacting with and inhibiting miR-382-3p to activate PD-L1 expression, resulting in immune escape [84]. In addition, LINC00473 deletion via transfection with siRNA inhibited PC progression and activated the killing capacity of CD8 + T cells by promoting miR-195-5p-targeted downregulation of PD-L1 and by increasing Bax, IFN-γ and IL-4 expression and decreasing Bcl-2, MMP-2, MMP-9 and IL-10 expression [85].

### Lung cancer

Accumulated evidence demonstrates that multiple lncRNAs are significantly associated with lung tumorigenesis and the immunotherapeutic response of lung cancer. Of note, among an established ceRNA network with 14 immune-related lncRNAs in LAD, lncRNA C5orf64 was found to have a positive relationship with the expression of immune molecules, including PD-1, PD-L1 and CTLA-4, and immune cells (M2 macrophages, monocytes, eosinophils and neutrophils) but was negatively correlated with Tregs and plasma cells [86], indicating a sole connection between lncRNAs and PD-1/PD-L1 levels. Similarly, lncRNA FGD5-AS1 functions as a sponge of miR-142 to regulate PD-L1 expression, leading to promotion of cell resistance to cisplatin (DDP) and cell proliferation, migration and invasion in LAD [87]. In contrast, the antisense lncRNA NKX2-1-AS1 downregulated PD-L1 expression by modulating NKX2-1 protein and decreased the expression of cell adhesion molecules, consequently inhibiting cell migration and wound healing in LAD [88]. Therefore, decreasing FGD5-AS1 or increasing NKX2-1-AS1 may be a promising strategy in PD-1/PD-L1 immunotherapy for patients with LAD. In NSCLC, lncRNA MALAT1 increased PD-L1 expression by sponging miR-200a-3p, consequently promoting NSCLC progression [89]. Furthermore, ZFPM2-AS1 attenuated ZFPM2 expression and was positively associated with PD-L1 expression, thus promoting the proliferation, migration, and invasion of NSCLC cells via modulation of the JAK-STAT and AKT pathways [90].

### Prostate cancer (PCa)

PCa is a global disease with high morbidity and mortality that threatens male health. Although androgen deprivation is an effective treatment and the main choice for therapeutic management of advanced PCa patients, it shows no effect on CRPC. CRPC patients also present de novo resistance to PD-1/PD-L1 axis blockade. Hence, more attention has been given to understanding the underlying mechanisms of PD-1/PD-L1 axis blockade. In PCa, several lncRNAs have been uncovered to contribute to malignant progression and PD-1/PD-L1 immunosuppression [91]. For example, lncAMPC increases PD-L1 expression to show immunosuppressive activities through the LIF/LIFR-stimulated JAK1-STAT3 pathway. In detail, lncAMPC promotes LIF expression in the cytoplasm by directly binding to and inactivating miR-637, and enhances LIFR transcription in the nucleus by decoying histone H1.2 away from the upstream sequence of the LIFR gene [92]. Furthermore, lncRNA KCNQ1OT1 in PCa cells was uncovered to contribute to inhibition of CD8 + T cell cytotoxicity and to induce malignant progression in PCa [91]. Notably, KCNQ1OT1 directly binds to and decreases miR-15a and leads to recovery of PD-L1 expression, consequently inhibiting cytotoxicity and proliferation and promoting apoptosis of CD8 + T cells while promoting viability, migration, invasion, EMT and suppressing apoptosis of PCa cells [91].

### Bladder cancer

To date, very few studies have focused on the lncRNA-mediated PD-L1 expression. In one study, knockout of lncRNA UCA1 was found to promote apoptosis of 5637 bladder cancer cells; activate DCs; induce cytokine secretion, including IL-6, IL-12, IL-23 and TNFα; and more importantly, elevate PD-L1 expression [93]. Accordingly, cotreatment with anti-PD-1 and anti-UCA1 retarded tumor growth and increased the survival of xenografted mice, demonstrating synergistic efficacy in bladder cancer [93]. Another research group established a machine learning-based computational framework and found several lncRNA signatures of TIL-Bs, including TNRC6C-AS1, WASIR2, GUSBP11, OGFRP1, AC090515.2, PART1, MAFG-DT and LINC01184 [94]. Further investigation is warranted to determine the role of these lncRNAs in immunotherapy for bladder cancer.

### Breast cancer

LncRNAs XIST, TSIX, GATA3-AS1 and LINK-A play an oncogenic role in tumor progression and immune evasion in breast cancer and are all notably upregulated in breast cancer tissues, which is related to the expression of PD-L1 [95–97]. XIST and TSIX are overexpressed in the lymph nodes, and different body fluids of breast cancer patients but differentially expressed in patients with different breast cancer subtypes [95]. LncRNA GATA3-AS1 is highly expressed in TNBC tissues and plays an oncogenic role in tumor progression and immune evasion [97]. More importantly, GATA3-AS1 increases the expression of PD-L1 protein and decreases GATA3 expression to induce immune evasion by regulating the miR-676-3p/COPS5 pathway [97]. Furthermore, patients with TNBC resistant to PD-1 blockade therapy present a high expression level of lncRNA LINK-A and a low level of antigen PLC components [96]. In addition, LINK-A expression decreased the antigen PLC and intrinsic tumor suppressors Rb and p53 via K48-polyubiquitination [96]. LncRNA TCL6 has significant positive relationships with immune checkpoint molecules, including PD-1, PD-L1, PD-L2, and CTLA-4, as well as with immune infiltrating cells, such as B cells, CD8 + T cells, CD4 + T cells, neutrophils, and DCs, predicting poor survival in breast cancer [98].

### Thymoma

Thymomas are a group of rare neoplasms that occur in the anterior mediastinum. Pseudogene RP11-424C20.2, which can predict a better prognosis in thymoma, acts as a ceRNA to enhance UHRF1 expression by sponging miR-378a-3p, consequently regulating IFN-γ-mediated CLTA-4 and PD-L1 activity in thymomas [81]. To study the influence of lncRNA XLOC_003810 on the PD-1/PD-L1 pathway in MG-T cells, one group found that MG and MG-T group tissues displayed higher XLOC_003810 expression than control group tissues and had an increased frequency of CD4 + T cells and higher production of inflammatory cytokines. In addition, XLOC_003810 increases T cell activation and blocks the PD-1/PD-L1 pathway in MG-T patients [99]. Furthermore, lncRNA IFN-stimulated noncoding RNA 1 (INCR1), transcribed from the PD-L1 locus, is correlated with PD-L1 expression levels in tissues and is involved in regulation of tumor IFNγ signaling. IFNγ is often secreted by activated T cells and regulates immune responses and tumor immunosurveillance [100]. In detail, downregulation of INCR1 in several tumor cell lines led to suppression of PD-L1 expression, and tumor spheres with INCR1 downregulation were more sensitive to CD8 + T cell-mediated killing. Antisense oligonucleotides that disrupt the binding between INCR1 and HNRNPH1 suppressed PD-L1 and JAK2 expression [101].

### Head and neck squamous cell carcinoma (HNSCC)

By analyzing the expression of lncRNA AC131097.3 and PD-1 in HNSCC tissues and normal adjacent tissues from TCGA and in isolated leukocytes, it was noticed that lncRNA AC131097.3 and PD-1 are coexpressed, and that both of them are inversely correlated with promoter methylation and positively correlated with CpG methylation [102]. This result suggests that the DNA methylation landscape of AC131097.3 and PD-1 may contribute to HNSCC carcinogenesis. Furthermore, lncMX1-215 is upregulated by IFNα and inhibits proliferation and metastasis of HNSCC cells. Interestingly, lncMX1-215 negatively regulated PD-L1 expression to inhibit immune escape by suppressing H3K27 acetylation via binding to GCN5, an H3K27 acetylase [103].

### Nasopharyngeal carcinoma (NPC)

Using whole genome expression profiling data in NPC samples, AFAP1-AS1 was found to be correlated with PD-1 expression. Further study identified the coexpression of AFAP1-AS1 and PD-1 in infiltrating lymphocytes in NPC samples, and patients with positive expression of both AFAP1-AS1 and PD-1 had the poorest prognosis [104], suggesting that coexpression of AFAP1-AS1 and PD-1 may be an ideal target for future clinical trials of anti-PD-1 immune therapy. In addition, lncRNA HOXA-AS2 significantly promoted proliferation, invasion and migration of NPC cells by upregulating HIF-1α and PD-L1 expression via direct targeting miR-519 [105]. Consistently, a miR-519 inhibitor rescued HOXA-AS2 knockdown-attenuated progression of NPC [105].

### Melanoma

In skin cutaneous melanoma, lncRNA MIR155HG was found to be correlated with better survival of patients and with levels of infiltrating immune cells and immune molecules, such as PD-1 and PD-L1 [76]. Higher expression of MIR155HG was associated with poor overall survival in uveal melanoma, which was also correlated with PD-1, PD-L1 and CTLA4 [76]. In another study analyzing a public transcriptomic database of melanoma patients treated with anti-PD-1 monotherapy, a 15 lncRNA signature consisting of AC010904.2, LINC01126, AC012360.1, AC024933.1, AL442128.2, AC022211.4, AC022211.2, AC127496.5, NARF-AS1, AP000919.3, AP005329.2, AC023983.1, AC023983.2, AC139100.1, and AC012615.4, was identified as a significant biomarker for predicting prognosis in advanced melanoma patients, who were treated with anti-PD-1 monotherapy (nivolumab or pembrolizumab) [106]. These reports suggest that lncRNAs participate in regulation of the PD-1/PD-L1 pathway in melanoma. However, related studies are limited, and more studies are needed to explore the relationship between lncRNAs and the PD-1/PD-L1 pathway in melanoma.

### Other cancers

LncRNA SNHG20 is highly expressed in ESCC and significantly correlated with tumor size, grade, TNM stage and lymph node metastasis. SNHG20 regulates PD-L1 expression and promotes the proliferation, migration, invasion, and EMT of ESCC cells through modulation of the ATM/JAK pathway [107]. LncRNA RP11-571M6.8 plays an important role in immune evasion and indicates poor survival in GBM. In detail, RP11-571M6.8 is closely related to the expression of PD-1, PD-L1 and CTLA-4, as well as to regulatory T cell infiltration levels and their markers (IL2RA and FCGR2B) [108]. In ATC, UCA1 positively regulates PD-L1 expression by inhibiting miR-148a, consequently suppressing the killing effect of cytotoxic CD8 + T cells and reducing cytokine secretion [109]. MIR17HG was found to promote CRC tumorigenesis and metastasis and induce upregulation of PD-L1 by competitively sponging miR-375 to increase NF-κB/RELA expression [110]. Another group analyzed the extensive molecular characterization of 228 cervical cancer patients, and identified amplifications in PD-L1, PD-L2, and lncRNA BCAR4, which are related to the response to lapatinib [111]. In summary, these results suggest that the lncRNA-miRNA network in regulation of the PD-1/PD-Ls pathway could contribute to carcinogenesis and immune evasion (Table 1).

**Table 1:** LncRNAs in the regulation of PD-1/PD-L1 pathway

LncRNA	Cancer	Functions	Sponge miRNA	Targets	PD-1/PD-L1	Ref
SNHG14	DLBCL	Promotes tumorigenesis and immune evasion	miR-5590-3p	ZEB1	Activates PD-1/PD-L1	[61]
MALAT1		Promotes tumorigenesis and immune escape, inactivates CD8 + T cells	miR-195	N/S	Increases PD-L1	[62]
EMX2OS	OC	Promotes tumorigenesis	miR-654	AKT3	Increases PD-L1	[65]
HOTTIP		Promotes immune escape and inactivates T cell immunity	N/S	c-Jun	Increases PD-L1	[67]
SNHG12		Promotes immune escape	miR-21	IL-6	Increases PD-L1	[68]
Lnc-OC1	EC	Promote tumorigenesis	miR-34a	N/S	Increases PD-L1	[69]
MEG3	EC	Inhibits tumorigenesis	miR-216a	N/S	Increases PD-L1	[70]
BCAR4	CC	Regulates the response to lapatinib	N/S	N/S	Increases PD-L1, PD-L2	[111]
SNHG15	GC	Promotes tumorigenesis and immune escape	miR-141	N/S	Increases PD-L1	[73]
HIF1A-AS2		Promotes tumorigenesis and immune escape	miR-429	N/S	Increases PD-L1	[75]
NUTM2A-AS1		Promotes tumorigenesis, immune escape and drug resistance	miR-376a	TET1, HIF-1A	Increases PD-L1	[74]
MIAT	HCC	Promotes immune escape, regulate sensitivity to sorafenib	N/S	AK2, SLC6A6, KCND1, MEIS3, RIN1	Increases PD-1, PD-L1 and CTLA4	[79]
CASC11		Promotes cell proliferation, mobility, and glucose metabolism	N/S	NF-κB, PI3K/AKT/mTOR	Increases PD-L1	[80]
PCED1B-AS1		Promotes immune escape, inactivates receipt T cells and macrophages	hsa-mir-194-5p	N/S	Increases PD-L1, PD-L2	[77]
RP11-424C20.2I		Promotes immune escape	miR-378a-3p	UHRF1	Increases CLTA-4, PD-L1	[81]
XIST			miR-194-5p, miR-155-5p	N/S	Increases PD-L1	[83]
KCNQ1OT1		Promotes sorafenib resistance and immune escape	miR-506	N/S	Increases PD-L1	[82]
PMSB8-AS1	PC	Promotes proliferation, invasion, and migration	miR-382-3p	STAT1	Increases PD-L1	[84]
INC00473		Induces cancer progression and inactivate CD8+ T cells	miR-195-5p	Bax, IFN-γ, IL-4; Bcl-2, MMP-2, MMP-9, IL-10	Increases PD-L1	[85]
MIR17HG	CRC	Promotes tumorigenesis and metastasis	miR-375	NF-κB/RELA	Increases PD-L1	[110]
lncRNA C5orf64	LAD		N/S	N/S	Increases PD-1, PD-L1, CTLA-4, and immune cells	[86]
FGD5-AS1		Promotes tumorigenesis and resistance to cisplatin	miR-142	N/S	Increases PD-L1	[87]
NKX2-1-AS1		Inhibits cell migration	N/S	NKX2-1, cell adhesion molecules	Decreases PD-L1	[88]
MALAT1	NSCLC	Promotes cancer progression	miR-200a-3p	N/S	Increases PD-L1	[89]
ZFPM2-AS1		Promotes tumorigenesis	N/S	ZFPM2, JAK-STAT, Akt	Increases PD-L1	[90]
lncAMPC	PCa	Promotes malignant progression and immunosuppression	miR-637	Jak1-STAT3,LIF/LIFR	Increases PD-L1	[92]
KCNQ1OT1		Promotes malignant progression and inactivate CD8 + T cells	miR-15a	N/S	Increases PD-L1	[91]
UCA1	BLC	Promotes tumor progression, activate DCs and cytokines			Decreases PD-L1	[93]
GATA3-AS1	TNBC	Promotes cell progression and immune evasion	miR-676-3p	COPS5	Increases PD-L1, decreases GATA3	[97]
LINK-A	TNBC	Resistant to PD-1 blockade		PLC, Rb, p53		[96]
TCL6	BC	Promotes immune cell infiltration, related to poor survival	N/S	N/S	Increases PD-1, PD-L1, PD-L2, CTLA-4	[98]
RP11-424C20.2	Thy	Indicates a better prognosis regulate infiltrating immune cell	miR-378a-3p	UHRF1	CLTA-4 and PD-L1	[81]
XLOC_003810	MG-T	promotes T cell activation			inhibits PD-1/PD-L1	[99]
INCR1		Inhibits immune responses		IFN gamma signaling	Increases PD-L1	[101]
AC131097.3	HNSCC	Promotes carcinogenesis		DNA methylation	Increases PD-1	[102]
lncMX1-215		Inhibits immune escape, cell proliferation and metastasis		H3K27 acetylation	Decreases PD-L1	[103]
AFAP1-AS1	NPC	Metastasis and poor prognosis				[104]
HOXA-AS2		Promotes cell proliferation, invasion, migration	miR-519	HIF-1α	Increase PD-L1	[105]
SNHG20	ESCC	Promotes cell proliferation, migration, invasion, EMT	N/S	ATM/JAK	Increase PD-L1	[107]
RP11-571M6.8	GBM	Promotes immune evasion, indicates a poor survival	N/S	N/S	PD-1, PD-L1, CTLA-4	[112]
UCA1	ATC	Inactivates cytotoxic CD8 + T cells, inhibit cytokine secretion	miR-148a	N/S	Increase PD-L1	[109]

*BC* Breast cancer, *BLC* Bladder cancer, *DLBCL* Diffuse large B cell lymphoma, *CC* Cervical cancer, *CRC* Colorectal cancer, *OC* Ovarian cancer, *ZEB1* Zinc finger E-box binding homeobox 1, *EC* Endometrial cancer, *GBM* Glioblastoma multiforme, *PC* Pancreatic cancer, *LAD *Lung adenocarcinoma, *MG-T* Myasthenia gravis-related thymoma, *THY* Thymomas, *TNBC* Triple-negative breast cancer, *MG-T* Myasthenia gravis-related thymoma, *HNSCC* Head and neck squamous cell carcinoma

### CircRNAs regulate PD-1/PD-L1 pathway

Current research has demonstrated that circRNAs function as ceRNAs to regulate PD-L1 expression, thereby regulating tumor immune escape in many tumors. Tumor immune escape is an important process in the survival and invasion of tumor cells by which cancer cells grow and metastasize by avoiding recognition and attack by the immune system. Many circRNAs are reported to act as key factors by regulating the PD-1/PD-L1 pathway to influence the TME.

In several studies, dysregulation of circRNAs has been found to be related to the proliferation, migration, invasion and immune escape of lung cancer cells [113]. For example, one group used RT-qPCR analysis to assess blood samples from 231 lung cancer patients and 41 controls, and the results revealed that hsa_circ_0000190 overexpression was more likely to exist in patients with a larger tumor size, later stage, worse histological type, more distant metastatic organs, extrathoracic metastasis, and poor survival and prognosis [114]. Moreover, elevated hsa_circ_0000190 levels in plasma were associated with higher PD-L1 levels in tumors. At the same time, patients with higher plasma hsa_circ_0000190 levels tend to have poor responses to systemic therapy and immunotherapy [114].

Similarly, circFGFR1 derived from FGFR1 shows higher expression in NSCLC tissues, and its high expression is related to poor prognosis and clinicopathological features [115]. In addition, circFGFR1 can sponge miR-381-3p to enhance expression of the target gene CXCR4, and knockout of CXCR4 sensitized NSCLC cells to anti-PD-1 immunotherapy. Consequently, this circRNA elevated the proliferation and immune evasion of NSCLC cells and promoted resistance to anti-PD-1-based therapy [115]. CXCR4 can bind CXCL12 (stromal cell-derived factor-1), resulting in an increase in intracellular calcium, proliferation, cell adhesion, and gene transcription [116]. Consistently, circRNA circ-CPA4 (hsa_circ_0082374) functions as an oncogene in several tumor types, including glioma [117] and NSCLC [118]. Hong et al. revealed that circ-CPA4 and PD-L1 were overexpressed while let-7 miRNA was expressed at lower levels in NSCLC cells and tissues in comparison with normal bronchial epithelial (HBE) cells and adjacent tissues, respectively [118]. In addition, NSCLC patients with decreased expression of circ-CPA4 and PD-L1 and increased let-7 expression had a better prognosis. Mechanistically, circ-CPA4 targets and attenuates let-7 miRNA expression, and then upregulates PD-L1 expression to promote tumorigenesis and EMT in NSCLC cells. Remarkably, circ-CPA4 positively regulated exosomal PD-L1 levels. PD-L1 on the NSCLC cell membrane was found to paralyze CD8 + T cells, and in coculture of NSCLC cells and CD8 + T cells, removing circ-CPA4 reactivated CD8 + T cells, suggesting that circ-CPA4 enhanced PD-L1 expression by sponging let-7 to regulate cell growth, mobility, stemness and drug resistance and to inactivate CD8 + T cells in the TME in NSCLC.

Circ_0000284 has been validated as an oncogene in cholangiocarcinoma [112, 119] and NSCLC [120]. In cholangiocarcinoma, circ-0000284 was evidently elevated in cell lines, tumor tissues and plasma exosomes, and downregulation of circ-0000284 suppressed cell proliferation, migration and invasion and promoted apoptosis. Further RIP and dual-luciferase reporter assays demonstrated that circ-0000284 targeted miR-637 to elevate LY6E expression [112], thereby promoting immune escape. Consistently, elevated expression of circ-0000284 is associated with promotion of cancer cell migration, invasion and proliferation, and contributes to a poor prognosis in NSCLC patients. Importantly, circ_0000284 increases PD-L1 expression as a ceRNA of miR-377, leading to NSCLC development [120].

Another group uncovered that hsa_circ_0020397 was increased in CRC cells and negatively related to miR-138 expression. Circ_0020397 is capable of promoting CRC development by inactivating miR-138 to increase PD-L1 and TERT expression [121]. Furthermore, circRNA CDR1-AS was also found to be a tumor promotor and was closely linked with poor prognosis in colon cancer [122]. Tanaka et al. used a lactase 2 gene cassette to establish a colon cancer cell line with stable expression of CDR1-AS. SW620 cells overexpressing CDR1-AS showed significant upregulation of two important PD-L1 modulators, CMTM4 and CMTM6, suggesting that overexpression of CDR1-AS in cancer cells could enhance PD-L1 expression levels on the plasma membrane of tumor cells. However, this research group identified that CDR1-AS functions in regulation of PD-L1 independent of miR-7 [122].

In melanoma, circ_0020710, derived from CD151, was shown to have a high expression level in cancer tissues and was related to the malignant phenotype and poor prognosis of melanoma patients [123]. Furthermore, circ_0020710 plays a tumor-promoting role in melanoma by elevating cell proliferation, migration and invasion in vitro and tumor growth in vivo, and promoting immune evasion [123]. Mechanistically, circ_0020710 recruits immune suppressor cells to induce an immunosuppressive microenvironment via the promotion of the CXCL12/CXCR4/CXCR7 axis by sponging miR-370-3p. This process leads to the inhibition of cytotoxic lymphocyte exhaustion [123]. Hence, AMD3100, a CXCL12/CXCR4 axis inhibitor, could promote the antitumor efficacy of anti-PD-1 treatment in melanoma cells [123]. In pancreatic cancer, another study revealed that circ-UBAP2 and hsa-miR-494 can modulate the expression of CXCR4, HIF1A, ZEB1, and SDC1. CXCR4 and ZEB1 expression is positively associated with the expression of CTLA-4 and PD-1, indicating that circ-UBAP2 might repress antigen presentation and promote immune escape in pancreatic cancer [124]. Based on the above findings, circRNAs may function as oncogenes or tumor suppressors and regulate PD-L1 expression to govern the progression and immune evasion of cancer by regulating target genes via miRNA sponging (Table 2).

**Table 2:** CircRNAs in the regulation of PD-1/PD-L1 pathways

CircRNA	Cancer	Functions	Sponge miRNA	Targets	PD-1/PD-L1	Reference
Hsa_circ_0000190	NSCLC	Indicates poor survival and prognosis	N/S	N/S	Increases PD-L1	[114]
CircFGFR1		Indicates poor prognosis and survival	miR-381-3p	CXCR4	Resistance to anti-PD-1 immunotherapy	[115]
Circ-CPA4		Indicates poor prognosis, inactives CD8 + T cells	let-7	N/S	Increases PD-L1	[118]
Circ_0000284		Induces cell migration, invasion and proliferation	miR-377	N/S	Increases PD-L1	[120]
Hsa_circ_0020397	CRC	Induces cell viability and invasion, inhibits cell apoptosis	miR-138	TERT	Increases PD-L1	[121]
CDR1-AS	CRC	Related to poor prognosis	Independent on miR-7	CMTM4, CMTM6	Increases PD-L1	[122]
Circ_0020710	Mel	Induces cell proliferation, migration, invasion; inhibits cytotoxic lymphocyte exhaustion	miR-370-3p	CXCL12/CXCR4/CXCR7	Immune suppressive	[123]
Circ-UBAP2	PAAD	Inhibits antigen presentation; induces immune escape	miR-494	CXCR4 and ZEB1	Increases CTLA-4 and PD-1	[124]

*Mel* Melanoma, *NSCLC* Non-small-cell lung cancer, *PAAD* Pancreatic adenocarcinoma

### LncRNAs and circRNAs regulate TME-driven immune evasion

Several mechanisms of immune escape include defective antigen presentation, alterations in tumor death pathways, abnormal metabolism, and recruitment of immunosuppressive cells and abnormal cytokines in the TME [125]. The TME is a complex scaffold of stromal and epithelial cells, including tumor cells, immune cells, extracellular matrix (ECM) components and exosomes, which critically participate in immune evasion [126]. For instance, immune cells interacting with intercellular stromal cells led to immune evasion of malignant B cells [127]. In the TME, lncRNAs and circRNAs play an essential role in regulation of tumor progression and immunity.

Hypoxia, an important feature of the TME, has a great impact on cancer aggressiveness and therapy. For example, under hypoxic conditions, circDENND4C silencing inhibited glycolysis, migration and invasion of breast cancer cells by increasing miR-200b/c [128]. LncRNAs also serve as transcriptional targets of HIF and can transfer hypoxia responses between cancer cells and the TME [129]. In another study, it was observed that hypoxia could induce circ-0000977 expression and enhance the HIF1α-mediated immune escape of PC cells by regulating miR-153 and its two targets HIF1α and ADAM10 [130]. PD-L1 was reported to be a direct target of HIF-1α, and blockade of PD-L1 promoted MDSC-involved T cell activation under hypoxia [37].

LncRNAs and circRNAs have an effect on various immune cells within the TME [131, 132]. Immune cells include myeloid cells, macrophages, dendritic cells, lymphoid cells and T cells. LncRNA LNMAT1 can enhance the interaction between hnRNPL and the CCL2 promoter, leading to macrophage recruitment into the tumor mass and elevating lymphatic metastasis in bladder cancer [133]. LncRNA lnc-BM enhanced crosstalk between macrophages and breast tumor cells, resulting in promotion of breast cancer brain metastasis in the brain TME [134]. Depletion of XIST in breast cancer induced M1-M2 macrophage polarization of microglia, leading to promotion of immunosuppressive cytokines and blockade of T-cell proliferation [135]. A decrease in lncRNA CCAT1 induced M2 macrophage polarization and cell migration by inhibiting PKCζ through the upregulation of miR-148A in prostate cancer [136]. LncRNA FENDRR suppressed Treg-induced immune evasion by sponging miR-423-5p and increasing GADD45B in HCC cells [137]. In the TME, some immunosuppressive cytokines can regulate the expression of lncRNAs in cancer cells, contributing to immune evasion. The cytokine IL-6 in the TME increased the expression of lncTCF7 and facilitated HCC aggressiveness via activation of STAT3 and EMT [138]. M2-like TAMs secrete EGF and enhance tumor metastasis by inhibiting lncRNA LIMT expression and activating the EGFR-ERK pathway in ovarian cancer [139]. In addition, immune cell-derived lncRNAs also regulate immune escape in the TME, including lymphoid immune cells, TAMs, and MDSCs [132]. One recent review summarized that lncRNAs regulate immune cell differentiation and function and are involved in immune evasion [140]. Taken together, lncRNAs and circRNAs affect the TME to shape the tumor suppressive microenvironment, supporting the development and malignancy of tumors.

## Conclusion and perspective

**Fig. 2:**
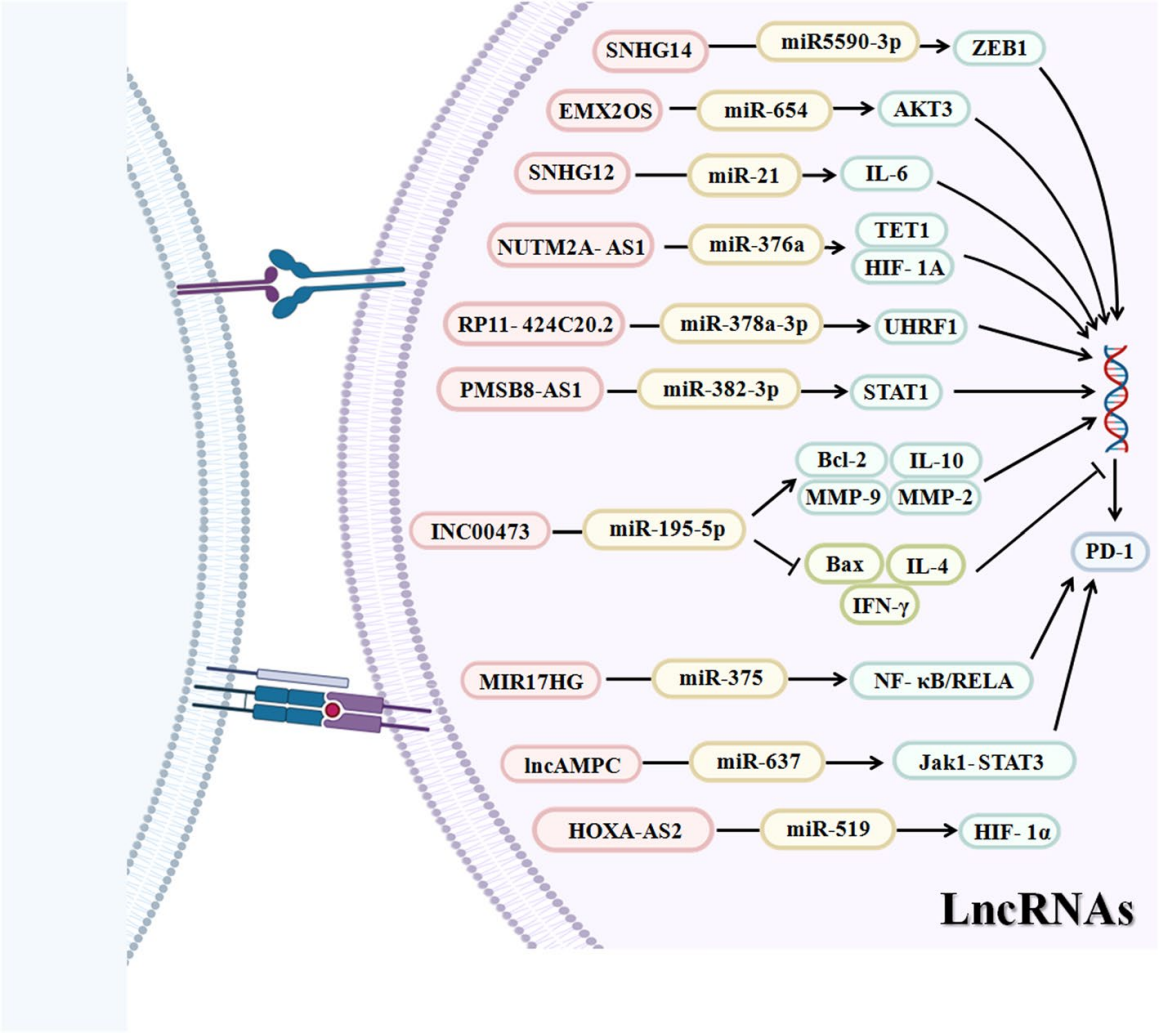
Multiple lncRNAs regulate the PD-1/PD-L1 pathway in cancer

**Fig. 3:**
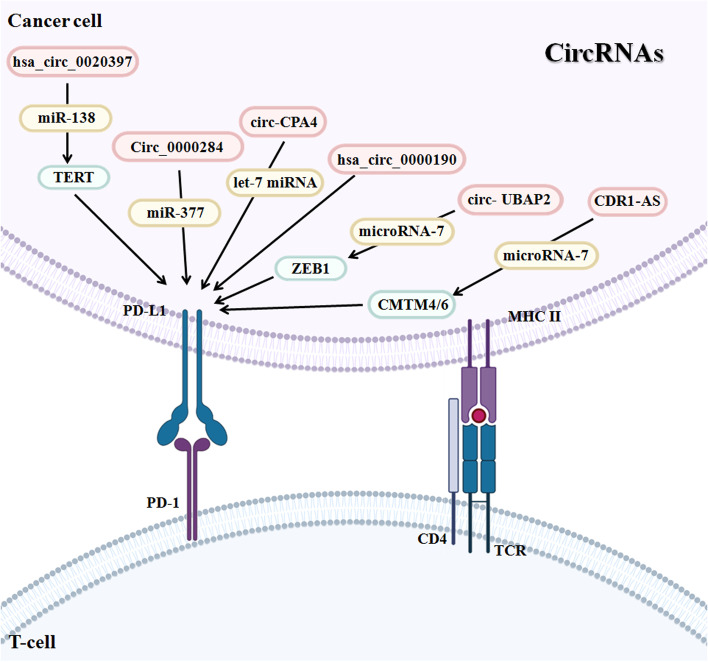
Several circRNAs regulate the PD-1/PD-L1 pathway in cancer

In summary, lncRNAs and circRNAs are critically involved in regulating the PD-1/PD-L1 pathway, resulting in participation in the immune response and immunotherapy (Figs. 2 and 3). Due to the pivotal role of lncRNAs in regulating the PD-1/PD-L1 pathway, the application of lncRNAs as immunotherapy targets could be a useful strategy for enhancing treatment efficiency in cancer patients. Although lncRNAs and circRNAs play a pivotal role in the immune response by targeting PD-1/PD-L1, several issues need to be addressed to fully understand the functions and mechanisms of noncoding RNA-mediated regulation of the PD-1/PD-L1 pathway. Several clinical trials are ongoing to explore whether lncRNAs, including CCAT1, HOTAIR, and H19, can be considered potential biomarkers in lung cancer (NCT03830619), colorectal cancer (NCT04269746), thyroid cancer (NCT03469544), HCC (NCT04767750), and stomach cancer (NCT03057171). To date, no study or clinical trials are available to determine whether lncRNAs and circRNAs are potential targets to regulate PD-1/PD-L1 in cancer immunotherapy (clinicalTrials.gov). Compared with other upstream factors of PD-1/PD-L1, such as NOTCH, FOXO1 and IRF9, are noncoding RNAs more important to target the PD-1/PD-L1 pathway in cancer? Numerous of noncoding RNAs are involved in PD-1/PD-L1 regulation. Which noncoding RNA is paramount to control the PD-1/PD-L1 pathway? Two studies showed that linc00511-siRNA conjugated nanobubbles can improve linc00511 siRNA delivery and increase cisplatin sensitivity in TNBC cells [141, 142]. LncRNA MEG3 was also encapsulated into aptamer-functionalized dendrimer nanoparticles to treat castration-resistant prostate cancer [143]. Developing a delivery system to deliver noncoding RNAs to specific organs is a challenge. LncRNA MIR155HG is considered a prognostic biomarker in multiple cancer types [76]. LncRNAs XIST and TSIX were reported to act as cancer immune biomarkers in breast cancer patients with PD-L1 overexpression [95]. LncAMPC regulates immunosuppression and could work as a prognostic biomarker and therapeutic target in prostate cancer [92]. It is essential to determine whether more lncRNAs and circRNAs could serve as biomarkers and therapeutic targets. It is necessary to note that several reports have shown only an indirect association between PD-1/PD-L1 and lncRNAs, and not a direct regulation of PD-1/PD-L1 by lncRNAs in multiple cancer types, such as EC [69] and GC [72]. Therefore, the detailed molecular mechanisms of noncoding RNA-involved modulation of the PD-1/PD-L1 pathway are still not fully elucidated in multiple human cancers types. Thus, further investigations to address these concerns will help us understand the role of noncoding RNAs in the cancer immune response and in tumor immunotherapy.

## Data Availability

Not applicable.

## Abbreviations

AFAP1-AS1: Actin filament-associated protein 1 antisense RNA 1
APCs: Antigen presenting cells
ATC: Anaplastic thyroid carcinoma
Bcl-2: B-cell lymphoma-2
CASC11: Cancer susceptibility candidate 11
ceRNA: Competing endogenous RNA
CircRNAs: Circular RNAs
CircFGFR1: CircRNA fibroblast growth factor receptor 1
CPRC: Castration-resistant prostate cancer
CRC: Colorectal cancer
CTLA-4: Cytotoxic T lymphocyteassociated antigen 4
CXCR4: C-X-C motif chemokine receptor 4
DCs: Dendritic cells
EMT: Epithelial to mesenchymal transition
ENCODE: The encyclopedia of DNA elements
ESCC: Esophageal squamous cell carcinoma
FDA: Food and Drug Administration
FGD5-AS1: FGD5-antisense 1
FOXO1: Forkhead box protein O1
GBM: Glioblastoma multiforme
HIF1A-AS2: Hypoxia-inducible factor-1 alpha antisense RNA-2
HIF-1α: Hypoxia-inducible factor-1alpha
HOXA-AS2: HOXA cluster antisense RNA 2
HOTTIP: HOXA transcript at the distal tip
ICB: Immune checkpoint blockade
IDO1: Indoleamine 2,3-dioxygenase 1
IFN-γ: Interferon-gamma
LAD: Lung adenocarcinoma
LIHC: Liver hepatocellular carcinoma
KCNQ1OT1: KCNQ1 overlapping transcript 1
LncRNAs: Long non-coding RNAs
lncAMPC: LncRNA activated in metastatic PCa
MALAT1: Metastasis-associated lung adenocarcinoma transcript 1
MDSCs: Myeloid-derived suppressor cells
MG-T: Myasthenia gravis-related thymoma
MHC: Major histocompatibility complexes
MMP-2: Matrix metalloproteinase 2
ncRNAs: Noncoding RNAs
NFAT: Nuclear factor of activated T cells
NK cells: Natural killer cells
NSCLC: Non-small cell lung cancer
PD-1: Programmed cell death-1
PLC: Peptide-loading complex
piRNAs: Piwi-interacting RNAs
SHP-2: Src homology region 2-containing protein tyrosine phosphatase 2
siRNAs: Small inhibitory RNAs
snRNAs: Small nuclear RNAs
snoRNAs: Small nucleolar RNAs
SNHG14: Small nucleolar RNA host gene 14
TAMs: Tumor associated macrophages
TCGA: The Cancer Genome Atlas
TME: Tumor microenvironment
TNBC: Triple-negative breast cancer
TCL6: T-cell leukemia/lymphoma 6
TIL-Bs: Tumor-infiltrating B lymphocytes
TLRs: Toll like receptors
TNF-α: Tumor necrosis factor-α
UCA1: Urothelial carcinoma associated 1
XCI: X chromosome inactivation
ZEB1: Zinc finger E-box binding homeobox 1
ZFPM2-AS1: Zinc finger protein multitype 2 antisense RNA 1

## References

[1] Abbott M, Ustoyev Y (2019). Cancer and the immune system: the history and background of immunotherapy. Semin Oncol Nurs.

[2] Salmaninejad A, Valilou SF, Shabgah AG, Aslani S, Alimardani M, Pasdar A, Sahebkar A (2019). PD-1/PD-L1 pathway: Basic biology and role in cancer immunotherapy. J Cell Physiol.

[3] Schneider H, Downey J, Smith A, Zinselmeyer BH, Rush C, Brewer JM, Wei B, Hogg N, Garside P, Rudd CE (2006). Reversal of the TCR stop signal by CTLA-4. Science.

[4] Ishida Y, Agata Y, Shibahara K, Honjo T (1992). Induced expression of PD-1, a novel member of the immunoglobulin gene superfamily, upon programmed cell death. EMBO J.

[5] Ljunggren HG, Jonsson R, Hoglund P (2018). Seminal immunologic discoveries with direct clinical implications: the 2018 nobel prize in physiology or medicine honours discoveries in cancer immunotherapy. Scand J Immunol.

[6] Feng M, Xiong G, Cao Z, Yang G, Zheng S, Song X, You L, Zheng L, Zhang T, Zhao Y (2017). PD-1/PD-L1 and immunotherapy for pancreatic cancer. Cancer Lett.

[7] Han Y, Liu D, Li L (2020). PD-1/PD-L1 pathway: current researches in cancer. Am J Cancer Res.

[8] Yang KN, Han W, Qin YJ, Chen LN (2019). Effects of different levels of soluble PD-L1 protein on the growth of Lewis lung cancer transplanted tumor. J Biol Regul Homeost Agents.

[9] Chang E, Pelosof L, Lemery S, Gong Y, Goldberg KB, Farrell AT, Keegan P, Veeraraghavan J, Wei G, Blumenthal GM, et al: Systematic Review of PD-1/PD-L1 Inhibitors in Oncology: From Personalized Medicine to Public Health. Oncologist. 2021. 10.1002/onco.13887. Online ahead of print. 10.1002/onco.13887 PMC8488782 34196068

[10] Lucibello G, Mograbi B, Milano G, Hofman P, Brest P: PD-L1 regulation revisited: impact on immunotherapeutic strategies. Trends Mol Med. 2021;S1471-4914(21)00152-0. 10.1016/j.molmed.2021.06.005. Online ahead of print. 10.1016/j.molmed.2021.06.005 34187739

[11] Tian Y, Huang A, Yang Y, Dang Q, Wen Q, Wang L, Sun Y (2021). Assessment of the clinical trials safety profile of pd-1/pd-l1 inhibitors among patients with cancer: an updated systematic review and meta-analysis. Front Oncol.

[12] Ding L, Lu S, Li Y (2020). Regulation of PD-1/PD-L1 pathway in cancer by noncoding RNAs. Pathol Oncol Res.

[13] Shek D, Read SA, Akhuba L, Qiao L, Gao B, Nagrial A, Carlino MS, Ahlenstiel G (2020). Non-coding RNA and immune-checkpoint inhibitors: friends or foes?. Immunotherapy.

[14] Skafi N, Fayyad-Kazan M, Badran B (2020). Immunomodulatory role for MicroRNAs: regulation of PD-1/PD-L1 and CTLA-4 immune checkpoints expression. Gene.

[15] Wang Q, Lin W, Tang X, Li S, Guo L, Lin Y, Kwok HF (2017). The roles of microRNAs in regulating the expression of PD-1/PD-L1 immune checkpoint. Int J Mol Sci.

[16] Keir ME, Butte MJ, Freeman GJ, Sharpe AH (2008). PD-1 and its ligands in tolerance and immunity. Annu Rev Immunol.

[17] Dong H, Zhu G, Tamada K, Chen L (1999). B7–H1, a third member of the B7 family, co-stimulates T-cell proliferation and interleukin-10 secretion. Nat Med.

[18] Neel BG, Gu H, Pao L. The ’Shp’ing news: SH2 domain-containing tyrosine phosphatases in cell signaling. Trends Biochem Sci. 2003;28:284–93. 10.1016/S0968-0004(03)00091-4 12826400

[19] Carreno BM, Collins M (2002). The B7 family of ligands and its receptors: new pathways for costimulation and inhibition of immune responses. Annu Rev Immunol.

[20] Barber DL, Wherry EJ, Masopust D, Zhu B, Allison JP, Sharpe AH, Freeman GJ, Ahmed R (2006). Restoring function in exhausted CD8 T cells during chronic viral infection. Nature.

[21] Gordon SR, Maute RL, Dulken BW, Hutter G, George BM, McCracken MN, Gupta R, Tsai JM, Sinha R, Corey D (2017). PD-1 expression by tumour-associated macrophages inhibits phagocytosis and tumour immunity. Nature.

[22] Lin X, Lu X, Luo G, Xiang H (2020). Progress in PD-1/PD-L1 pathway inhibitors: from biomacromolecules to small molecules. Eur J Med Chem.

[23] Staron MM, Gray SM, Marshall HD, Parish IA, Chen JH, Perry CJ, Cui G, Li MO, Kaech SM (2014). The transcription factor FoxO1 sustains expression of the inhibitory receptor PD-1 and survival of antiviral CD8(+) T cells during chronic infection. Immunity.

[24] Li C, Li W, Xiao J, Jiao S, Teng F, Xue S, Zhang C, Sheng C, Leng Q, Rudd CE (2015). ADAP and SKAP55 deficiency suppresses PD-1 expression in CD8+ cytotoxic T lymphocytes for enhanced anti-tumor immunotherapy. EMBO Mol Med.

[25] Youngblood B, Oestreich KJ, Ha SJ, Duraiswamy J, Akondy RS, West EE, Wei Z, Lu P, Austin JW, Riley JL (2011). Chronic virus infection enforces demethylation of the locus that encodes PD-1 in antigen-specific CD8(+) T cells. Immunity.

[26] Chen S, Crabill GA, Pritchard TS, McMiller TL, Wei P, Pardoll DM, Pan F, Topalian SL (2019). Mechanisms regulating PD-L1 expression on tumor and immune cells. J Immunother Cancer.

[27] Xu-Monette ZY, Zhou J, Young KH (2018). PD-1 expression and clinical PD-1 blockade in B-cell lymphomas. Blood.

[28] Ostrand-Rosenberg S, Horn LA, Haile ST (2014). The programmed death-1 immune-suppressive pathway: barrier to antitumor immunity. J Immunol.

[29] Zou MX, Lv GH, Wang XB, Huang W, Li J, Jiang Y, She XL (2019). Clinical impact of the immune microenvironment in spinal chordoma: immunoscore as an independent favorable prognostic factor. Neurosurgery.

[30] Liu P, Xiao Q, Zhou B, Dai Z, Kang Y (2019). Prognostic significance of programmed death ligand 1 expression and tumor-infiltrating lymphocytes in axial osteosarcoma. World Neurosurg.

[31] Ohaegbulam KC, Assal A, Lazar-Molnar E, Yao Y, Zang X (2015). Human cancer immunotherapy with antibodies to the PD-1 and PD-L1 pathway. Trends Mol Med.

[32] Shen X, Zhang L, Li J, Li Y, Wang Y, Xu ZX (2019). Recent findings in the regulation of programmed death ligand 1 expression. Front Immunol.

[33] Sharpe AH, Wherry EJ, Ahmed R, Freeman GJ (2007). The function of programmed cell death 1 and its ligands in regulating autoimmunity and infection. Nat Immunol.

[34] Magiera-Mularz K, Kocik J, Musielak B, Plewka J, Sala D, Machula M, Grudnik P, Hajduk M, Czepiel M, Siedlar M (2021). Human and mouse PD-L1: similar molecular structure, but different druggability profiles. iScience.

[35] Zou W, Wolchok JD, Chen L (2016). PD-L1 (B7-H1) and PD-1 pathway blockade for cancer therapy: Mechanisms, response biomarkers, and combinations. Sci Transl Med.

[36] Tang H, Liang Y, Anders RA, Taube JM, Qiu X, Mulgaonkar A, Liu X, Harrington SM, Guo J, Xin Y (2018). PD-L1 on host cells is essential for PD-L1 blockade-mediated tumor regression. J Clin Invest.

[37] Noman MZ, Desantis G, Janji B, Hasmim M, Karray S, Dessen P, Bronte V, Chouaib S (2014). PD-L1 is a novel direct target of HIF-1alpha, and its blockade under hypoxia enhanced MDSC-mediated T cell activation. J Exp Med.

[38] Sun C, Mezzadra R, Schumacher TN (2018). Regulation and Function of the PD-L1 Checkpoint. Immunity.

[39] Wherry EJ, Kurachi M (2015). Molecular and cellular insights into T cell exhaustion. Nat Rev Immunol.

[40] Zhao R, Song Y, Wang Y, Huang Y, Li Z, Cui Y, Yi M, Xia L, Zhuang W, Wu X, Zhou Y. PD-1/PD-L1 blockade rescue exhausted CD8+ T cells in gastrointestinal stromal tumours via the PI3K/Akt/mTOR signalling pathway. Cell Prolif. 2019;52(3):e12571. 10.1111/cpr.12571 PMC6536456 30714229

[41] Stutvoet TS, Kol A, de Vries EG, de Bruyn M, Fehrmann RS (2019). Terwisscha van Scheltinga AG, de Jong S: MAPK pathway activity plays a key role in PD-L1 expression of lung adenocarcinoma cells. J Pathol.

[42] O’Sullivan Coyne G, Madan RA, Gulley JL. Nivolumab: promising survival signal coupled with limited toxicity raises expectations. J Clin Oncol. 2014;32:986–8. 10.1200/JCO.2013.54.5996 PMC6624830 24590655

[43] Wilkinson E. Nivolumab success in untreated metastatic melanoma. Lancet Oncol. 2015;16. 10.1016/S1470-2045(14)71129-5 25638562

[44] Zhang JY, Yan YY, Li JJ, Adhikari R, Fu LW (2020). PD-1/PD-L1 Based Combinational Cancer Therapy: Icing on the Cake. Front Pharmacol.

[45] Djebali S, Davis CA, Merkel A, Dobin A, Lassmann T, Mortazavi A, Tanzer A, Lagarde J, Lin W, Schlesinger F (2012). Landscape of transcription in human cells. Nature.

[46] Consortium EP (2012). An integrated encyclopedia of DNA elements in the human genome. Nature.

[47] Anastasiadou E, Jacob LS, Slack FJ (2018). Non-coding RNA networks in cancer. Nat Rev Cancer.

[48] Esteller M (2011). Non-coding RNAs in human disease. Nat Rev Genet.

[49] Ashwal-Fluss R, Meyer M, Pamudurti NR, Ivanov A, Bartok O, Hanan M, Evantal N, Memczak S, Rajewsky N, Kadener S (2014). circRNA biogenesis competes with pre-mRNA splicing. Mol Cell.

[50] Gong R, Jiang Y (2020). Non-coding RNAs in Pancreatic Ductal Adenocarcinoma. Front Oncol.

[51] Akhade VS, Pal D, Kanduri C (2017). Long Noncoding RNA: Genome Organization and Mechanism of Action. Adv Exp Med Biol.

[52] Iyer MK, Niknafs YS, Malik R, Singhal U, Sahu A, Hosono Y, Barrette TR, Prensner JR, Evans JR, Zhao S (2015). The landscape of long noncoding RNAs in the human transcriptome. Nat Genet.

[53] Dangelmaier E, Lal A: Adaptor proteins in long noncoding RNA biology. Biochim Biophys Acta Gene Regul Mech. 2020;1863(4):194370. 10.1016/j.bbagrm.2019.03.003. 10.1016/j.bbagrm.2019.03.003 30951902

[54] Kristensen LS, Andersen MS, Stagsted LVW, Ebbesen KK, Hansen TB, Kjems J (2019). The biogenesis, biology and characterization of circular RNAs. Nat Rev Genet.

[55] Qu S, Liu Z, Yang X, Zhou J, Yu H, Zhang R, Li H (2018). The emerging functions and roles of circular RNAs in cancer. Cancer Lett.

[56] Xu J, Shi A, Long Z, Xu L, Liao G, Deng C, Yan M, Xie A, Luo T, Huang J (2018). Capturing functional long non-coding RNAs through integrating large-scale causal relations from gene perturbation experiments. EBioMedicine.

[57] Galaznik A,, Huelin R, Stokes M, Guo Y, Hoog M, Bhagnani T, Bell  J, Shou Y (2018). Systematic review of therapy used in relapsed or refractory diffuse large B-cell lymphoma and follicular lymphoma. Future Sci OA.

[58] Zhao CC, Jiao Y, Zhang YY, Ning J, Zhang YR, Xu J, Wei W, Kang-Sheng G (2019). Lnc SMAD5-AS1 as ceRNA inhibit proliferation of diffuse large B cell lymphoma via Wnt/beta-catenin pathway by sponging miR-135b-5p to elevate expression of APC. Cell Death Dis.

[59] Yan Y, Han J, Li Z, Yang H, Sui Y, Wang M (2016). Elevated RNA expression of long noncoding HOTAIR promotes cell proliferation and predicts a poor prognosis in patients with diffuse large B cell lymphoma. Mol Med Rep.

[60] Su W, Xu M, Chen X, Chen N, Gong J, Nie L, Li L, Li X, Zhang M, Zhou Q (2017). Long noncoding RNA ZEB1-AS1 epigenetically regulates the expressions of ZEB1 and downstream molecules in prostate cancer. Mol Cancer.

[61] Zhao L, Liu Y, Zhang J, Liu Y, Qi Q (2019). LncRNA SNHG14/miR-5590-3p/ZEB1 positive feedback loop promoted diffuse large B cell lymphoma progression and immune evasion through regulating PD-1/PD-L1 checkpoint. Cell Death Dis.

[62] Wang QM, Lian GY, Song Y, Huang YF, Gong Y. LncRNA MALAT1 promotes tumorigenesis and immune escape of diffuse large B cell lymphoma by sponging miR-195. Life Sci. 2019;231. 10.1016/j.lfs.2019.03.040 30898647

[63] Torre LA, Trabert B, DeSantis CE, Miller KD, Samimi G, Runowicz CD, Gaudet MM, Jemal A, Siegel RL (2018). Ovarian cancer statistics, 2018. CA Cancer J Clin.

[64] Balch C, Huang TH, Brown R, Nephew KP (2004). The epigenetics of ovarian cancer drug resistance and resensitization. Am J Obstet Gynecol.

[65] Duan M, Fang M, Wang C, Wang H, Li M (2020). LncRNA EMX2OS Induces Proliferation, Invasion and Sphere Formation of Ovarian Cancer Cells via Regulating the miR-654-3p/AKT3/PD-L1 Axis. Cancer Manag Res.

[66] Zou T, Wang PL, Gao Y, Liang WT (2019). Long noncoding RNA HOTTIP is a significant indicator of ovarian cancer prognosis and enhances cell proliferation and invasion. Cancer Biomark.

[67] Shang A, Wang W, Gu C, Chen C, Zeng B, Yang Y, Ji P, Sun J, Wu J, Lu W (2019). Long non-coding RNA HOTTIP enhances IL-6 expression to potentiate immune escape of ovarian cancer cells by upregulating the expression of PD-L1 in neutrophils. J Exp Clin Cancer Res.

[68] Qian M, Ling W, Ruan Z (2020). Long non-coding RNA SNHG12 promotes immune escape of ovarian cancer cells through their crosstalk with M2 macrophages. Aging (Albany NY).

[69] Liu Y, Chang Y, Cai YX (2020). Inhibition of Lnc-OC1 Induced Cell Apoptosis and Decreased Cell Viability by Releasing miR-34a and Inhibiting PD-L1 in Endometrial Carcinoma. Reprod Sci.

[70] Xu D, Dong P, Xiong Y, Chen R, Konno Y, Ihira K, Yue J, Watari H. PD-L1 Is a Tumor Suppressor in Aggressive Endometrial Cancer Cells and Its Expression Is Regulated by miR-216a and lncRNA MEG3. Front Cell Dev Biol. 2020;8:598205. 10.3389/fcell.2020.598205 PMC7755603 33363153

[71] He Y, Wang X (2020). Identification of molecular features correlating with tumor immunity in gastric cancer by multi-omics data analysis. Ann Transl Med.

[72] Chen T, Zhang C, Liu Y, Zhao Y, Lin D, Hu Y, Yu J, Li G (2019). A gastric cancer LncRNAs model for MSI and survival prediction based on support vector machine. BMC Genomics.

[73] Dang S, Malik A, Chen J, Qu J, Yin K, Cui L, Gu M (2020). LncRNA SNHG15 Contributes to Immuno-Escape of Gastric Cancer Through Targeting miR141/PD-L1. Onco Targets Ther.

[74] Wang J, Yu Z, Wang J, Shen Y, Qiu J, Zhuang Z (2020). LncRNA NUTM2A-AS1 positively modulates TET1 and HIF-1A to enhance gastric cancer tumorigenesis and drug resistance by sponging miR-376a. Cancer Med..

[75] Mu L, Wang Y, Su H, Lin Y, Sui W, Yu X, Lv Z: HIF1A-AS2 Promotes the Proliferation and Metastasis of Gastric Cancer Cells Through miR-429/PD-L1 Axis. Dig Dis Sci. 2021. 10.1007/s10620-020-06819-w. Online ahead of print. 10.1007/s10620-020-06819-w 33555514

[76] Peng L, Chen Z, Chen Y, Wang X, Tang N (2019). MIR155HG is a prognostic biomarker and associated with immune infiltration and immune checkpoint molecules expression in multiple cancers. Cancer Med.

[77] Fan F, Chen K, Lu X, Li A, Liu C, Wu B: Dual targeting of PD-L1 and PD-L2 by PCED1B-AS1 via sponging hsa-miR-194–5p induces immunosuppression in hepatocellular carcinoma. Hepatol Int. 2021;15(2):444–58. 10.1007/s12072-020-10101-6. 10.1007/s12072-020-10101-6 33219943

[78] Zhang Y, Zhang L, Xu Y, Wu X, Zhou Y, Mo J (2020). Immune-related long noncoding RNA signature for predicting survival and immune checkpoint blockade in hepatocellular carcinoma. J Cell Physiol.

[79] Peng L, Chen Y, Ou Q, Wang X, Tang N. LncRNA MIAT correlates with immune infiltrates and drug reactions in hepatocellular carcinoma. Int Immunopharmacol. 2020;89(Pt A):107071. 10.1016/j.intimp.2020.107071 33221703

[80] Song H, Liu Y, Li X, Chen S, Xie R, Chen D, Gao H, Wang G, Cai B, Yang X. Long noncoding RNA CASC11 promotes hepatocarcinogenesis and HCC progression through EIF4A3-mediated E2F1 activation. Clin Transl Med. 2020;10(7):e220. 10.1002/ctm2.220 PMC7643871 33252856

[81] Yang J, Zhang Y, Song H. A disparate role of RP11-424C20.2/UHRF1 axis through control of tumor immune escape in liver hepatocellular carcinoma and thymoma. Aging (Albany NY). 2019;11:6422–39. 10.18632/aging.102197 PMC6738438 31442209

[82] Zhang J, Zhao X, Ma X, Yuan Z, Hu M (2020). KCNQ1OT1 contributes to sorafenib resistance and programmed deathligand1mediated immune escape via sponging miR506 in hepatocellular carcinoma cells. Int J Mol Med.

[83] Atwa SM, Handoussa H, Hosny KM, Odenthal M, Tayebi HME (2020). Pivotal role of long non-coding ribonucleic acid-X-inactive specific transcript in regulating immune checkpoint programmed death ligand 1 through a shared pathway between miR-194-5p and miR-155-5p in hepatocellular carcinoma. World J Hepatol.

[84] Zhang H, Zhu C, He Z, Chen S, Li L, Sun C (2020). LncRNA PSMB8-AS1 contributes to pancreatic cancer progression via modulating miR-382-3p/STAT1/PD-L1 axis. J Exp Clin Cancer Res.

[85] Zhou WY, Zhang MM, Liu C, Kang Y, Wang JO, Yang XH (2019). Long noncoding RNA LINC00473 drives the progression of pancreatic cancer via upregulating programmed death-ligand 1 by sponging microRNA-195-5p. J Cell Physiol.

[86] Pang Z, Chen X, Wang Y, Wang Y, Yan T, Wan J, Wang K, Du J (2021). Long non-coding RNA C5orf64 is a potential indicator for tumor microenvironment and mutation pattern remodeling in lung adenocarcinoma. Genomics.

[87] Zhu F, Niu R, Shao X, Shao X (2021). FGD5AS1 promotes cisplatin resistance of human lung adenocarcinoma cell via the miR1425p/PDL1 axis. Int J Mol Med.

[88] Kathuria H, Millien G, McNally L, Gower AC, Tagne JB, Cao Y, Ramirez MI (2018). NKX2-1-AS1 negatively regulates CD274/PD-L1, cell-cell interaction genes, and limits human lung carcinoma cell migration. Sci Rep.

[89] Wei S, Wang K, Huang X, Zhao Z, Zhao Z (2019). LncRNA MALAT1 contributes to non-small cell lung cancer progression via modulating miR-200a-3p/programmed death-ligand 1 axis. Int J Immunopathol Pharmacol.

[90] Wang X, Tang J, Zhao J, Lou B, Li L. ZFPM2-AS1 promotes the proliferation, migration, and invasion of human non-small cell lung cancer cells involving the JAK-STAT and AKT pathways. PeerJ. 2020;8:e10225. 10.7717/peerj.10225 PMC7594634 33173620

[91] Chen QH, Li B, Liu DG, Zhang B, Yang X, Tu YL (2020). LncRNA KCNQ1OT1 sponges miR-15a to promote immune evasion and malignant progression of prostate cancer via up-regulating PD-L1. Cancer Cell Int.

[92] Zhang W, Shi X, Chen R, Zhu Y, Peng S, Chang Y (2020). Novel Long Non-coding RNA lncAMPC Promotes Metastasis and Immunosuppression in Prostate Cancer by Stimulating LIF/LIFR Expression. Mol Ther..

[93] Zhen S, Lu J, Chen W, Zhao L, Li X (2018). Synergistic Antitumor Effect on Bladder Cancer by Rational Combination of Programmed Cell Death 1 Blockade and CRISPR-Cas9-Mediated Long Non-Coding RNA Urothelial Carcinoma Associated 1 Knockout. Hum Gene Ther.

[94] Zhou M, Zhang Z, Bao S, Hou P, Yan C, Su J, Sun J: Computational recognition of lncRNA signature of tumor-infiltrating B lymphocytes with potential implications in prognosis and immunotherapy of bladder cancer. Brief Bioinform. 2021;22(3):bbaa047. 10.1093/bib/bbaa047. 10.1093/bib/bbaa047 32382761

[95] Salama EA, Adbeltawab RE, El Tayebi HM (2019). XIST and TSIX: Novel Cancer Immune Biomarkers in PD-L1-Overexpressing Breast Cancer Patients. Front Oncol.

[96] Hu Q, Ye Y, Chan LC, Li Y, Liang K, Lin A, Egranov SD, Zhang Y, Xia W, Gong J (2019). Oncogenic lncRNA downregulates cancer cell antigen presentation and intrinsic tumor suppression. Nat Immunol.

[97] Zhang M, Wang N, Song P, Fu Y, Ren Y, Li Z, Wang J. LncRNA GATA3-AS1 facilitates tumour progression and immune escape in triple-negative breast cancer through destabilization of GATA3 but stabilization of PD-L1. Cell Prolif. 2020;53(9):e12855. 10.1111/cpr.12855 PMC7507373 32687248

[98] Zhang Y, Li Z, Chen M, Chen H, Zhong Q, Liang L, Li B (2020). lncRNA TCL6 correlates with immune cell infiltration and indicates worse survival in breast cancer. Breast Cancer.

[99] Hu B, Niu L, Jiang Z, Xu S, Hu Y, Cao K. LncRNA XLOC_003810 promotes T cell activation and inhibits PD-1/PD-L1 expression in patients with myasthenia gravis-related thymoma. Scand J Immunol. 2020;92(1):e12886. 10.1111/sji.12886 32243615

[100] Ivashkiv LB (2018). IFNgamma: signalling, epigenetics and roles in immunity, metabolism, disease and cancer immunotherapy. Nat Rev Immunol.

[101] Mineo M, Lyons SM, Zdioruk M, von Spreckelsen N, Ferrer-Luna R, Ito H, Alayo QA, Kharel P, Giantini Larsen A, Fan WY, et al. Tumor Interferon Signaling Is Regulated by a lncRNA INCR1 Transcribed from the PD-L1 Locus. Mol Cell. 2020;78(6):1207–23. 10.1016/j.molcel.2020.05.015 PMC7377926 32504554

[102] Bockhorst C, Dietrich J, Vogt TJ, Stauber RH, Strieth S, Bootz F, Dietrich D, Vos L (2021). The DNA methylation landscape of PD-1 (PDCD1) and adjacent lncRNA AC131097.3 in head and neck squamous cell carcinoma. Epigenomics.

[103] Ma H, Chang H, Yang W, Lu Y, Hu J, Jin S (2020). A novel IFNalpha-induced long noncoding RNA negatively regulates immunosuppression by interrupting H3K27 acetylation in head and neck squamous cell carcinoma. Mol Cancer.

[104] Tang Y, He Y, Shi L, Yang L, Wang J, Lian Y, Fan C, Zhang P, Guo C, Zhang S (2017). Co-expression of AFAP1-AS1 and PD-1 predicts poor prognosis in nasopharyngeal carcinoma. Oncotarget.

[105] Wang S, You H, Yu S (2020). Long non-coding RNA HOXA-AS2 promotes the expression levels of hypoxia-inducible factor-1alpha and programmed death-ligand 1, and regulates nasopharyngeal carcinoma progression via miR-519. Oncol Lett.

[106] Zhou JG, Liang B, Liu JG, Jin SH, He SS, Frey B, Gu N, Fietkau R, Hecht M, Ma H, Gaipl US: Identification of 15 lncRNAs Signature for Predicting Survival Benefit of Advanced Melanoma Patients Treated with Anti-PD-1 Monotherapy. Cells. 2021;10(5):977. 10.3390/cells10050977 PMC8143567 33922038

[107] Zhang C, Jiang F, Su C, Xie P, Xu L: Upregulation of long noncoding RNA SNHG20 promotes cell growth and metastasis in esophageal squamous cell carcinoma via modulating ATM-JAK-PD-L1 pathway. J Cell Biochem. 2019. 10.1002/jcb.28444. Online ahead of print. 10.1002/jcb.28444 30767270

[108] Zhang Y, Liao G, Bai J, Zhang X, Xu L, Deng C (2019). Identifying Cancer Driver lncRNAs Bridged by Functional Effectors through Integrating Multi-omics Data in Human Cancers. Mol Ther Nucleic Acids..

[109] Wang X, Zhang Y, Zheng J, Yao C, Lu X: LncRNA UCA1 attenuated the killing effect of cytotoxic CD8 + T cells on anaplastic thyroid carcinoma via miR-148a/PD-L1 pathway. Cancer Immunol Immunother. 2021;70(8):2235–45. 10.1007/s00262-020-02753-y PMC10992874 33486611

[110] Xu J, Meng Q, Li X, Yang H, Xu J, Gao N, Sun H, Wu S, Familiari G, Relucenti M (2019). Long Noncoding RNA MIR17HG Promotes Colorectal Cancer Progression via miR-17-5p. Cancer Res.

[111] Cancer Genome Atlas Research N, Albert Einstein College of M, Analytical Biological S, Barretos Cancer H, Baylor College of M, Beckman Research Institute of City of H, Buck Institute for Research on A, Canada's Michael Smith Genome Sciences C, Harvard Medical S, Helen FGCC, et al: Integrated genomic and molecular characterization of cervical cancer. Nature 2017, 543:378–384. 10.1038/nature21386 PMC5354998 28112728

[112] Wang S, Hu Y, Lv X, Li B, Gu D, Li Y (2019). Circ-0000284 arouses malignant phenotype of cholangiocarcinoma cells and regulates the biological functions of peripheral cells through cellular communication. Clin Sci (Lond)..

[113] Di X, Jin X, Li R, Zhao M, Wang K (2019). CircRNAs and lung cancer: Biomarkers and master regulators. Life Sci.

[114] Luo YH, Yang YP, Chien CS, Yarmishyn AA, Ishola AA, Chien Y, Chen YM, Huang TW, Lee KY, Huang WC, et al: Plasma Level of Circular RNA hsa_circ_0000190 Correlates with Tumor Progression and Poor Treatment Response in Advanced Lung Cancers. Cancers (Basel). 2020;12(7):1740. 10.3390/cancers12071740 PMC7408140 32629833

[115] Zhang PF, Pei X, Li KS, Jin LN, Wang F, Wu J, Zhang XM (2019). Circular RNA circFGFR1 promotes progression and anti-PD-1 resistance by sponging miR-381-3p in non-small cell lung cancer cells. Mol Cancer..

[116] Chatterjee S, Behnam Azad B, Nimmagadda S (2014). The intricate role of CXCR4 in cancer. Adv Cancer Res.

[117] Peng H, Qin C, Zhang C, Su J, Xiao Q, Xiao Y, Xiao K, Liu Q (2019). circCPA4 acts as a prognostic factor and regulates the proliferation and metastasis of glioma. J Cell Mol Med.

[118] Hong W, Xue M, Jiang J, Zhang Y, Gao X (2020). Circular RNA circ-CPA4/ let-7 miRNA/PD-L1 axis regulates cell growth, stemness, drug resistance and immune evasion in non-small cell lung cancer (NSCLC). J Exp Clin Cancer Res.

[119] Louis C, Desoteux M, Coulouarn C (2019). Exosomal circRNAs: new players in the field of cholangiocarcinoma. Clin Sci (Lond).

[120] Li L, Zhang Q, Lian K (2020). Circular RNA circ_0000284 plays an oncogenic role in the progression of non-small cell lung cancer through the miR-377-3p-mediated PD-L1 promotion. Cancer Cell Int.

[121] Zhang XL, Xu LL, Wang F (2017). Hsa_circ_0020397 regulates colorectal cancer cell viability, apoptosis and invasion by promoting the expression of the miR-138 targets TERT and PD-L1. Cell Biol Int.

[122] Tanaka E, Miyakawa Y, Kishikawa T, Seimiya T, Iwata T, Funato K, Odawara N, Sekiba K, Yamagami M, Suzuki T (2019). Expression of circular RNA CDR1AS in colon cancer cells increases cell surface PDL1 protein levels. Oncol Rep.

[123] Wei CY, Zhu MX, Lu NH, Liu JQ, Yang YW, Zhang Y, Shi YD, Feng ZH, Li JX, Qi FZ, Gu JY (2020). Circular RNA circ_0020710 drives tumor progression and immune evasion by regulating the miR-370-3p/CXCL12 axis in melanoma. Mol Cancer.

[124] Zhao R, Ni J, Lu S, Jiang S, You L, Liu H, Shou J, Zhai C, Zhang W, Shao S (2019). CircUBAP2-mediated competing endogenous RNA network modulates tumorigenesis in pancreatic adenocarcinoma. Aging (Albany NY).

[125] Liu L, Wang Q, Qiu Z, Kang Y, Liu J, Ning S, Yin Y, Pang D, Xu S (2020). Noncoding RNAs: the shot callers in tumor immune escape. Signal Transduct Target Ther.

[126] Hinshaw DC, Shevde LA (2019). The Tumor Microenvironment Innately Modulates Cancer Progression. Cancer Res.

[127] Nicholas NS, Apollonio B, Ramsay AG (2016). Tumor microenvironment (TME)-driven immune suppression in B cell malignancy. Biochim Biophys Acta.

[128] Ren S, Liu J, Feng Y, Li Z, He L, Li L, Cao X, Wang Z, Zhang Y (2019). Knockdown of circDENND4C inhibits glycolysis, migration and invasion by up-regulating miR-200b/c in breast cancer under hypoxia. J Exp Clin Cancer Res.

[129] Kuo TC, Kung HJ, Shih JW (2020). Signaling in and out: long-noncoding RNAs in tumor hypoxia. J Biomed Sci.

[130] Ou ZL, Luo Z, Wei W, Liang S, Gao TL, Lu YB (2019). Hypoxia-induced shedding of MICA and HIF1A-mediated immune escape of pancreatic cancer cells from NK cells: role of circ_0000977/miR-153 axis. RNA Biol.

[131] Denaro N, Merlano MC, Lo Nigro C (2019). Long noncoding RNAs as regulators of cancer immunity. Mol Oncol.

[132] Luo Y, Yang J, Yu J, Liu X, Yu C, Hu J, Shi H, Ma X (2020). Long Non-coding RNAs: Emerging Roles in the Immunosuppressive Tumor Microenvironment. Front Oncol.

[133] Chen C, He W, Huang J, Wang B, Li H, Cai Q, Su F, Bi J, Liu H, Zhang B (2018). LNMAT1 promotes lymphatic metastasis of bladder cancer via CCL2 dependent macrophage recruitment. Nat Commun.

[134] Wang S, Liang K, Hu Q, Li P, Song J, Yang Y, Yao J, Mangala LS, Li C, Yang W (2017). JAK2-binding long noncoding RNA promotes breast cancer brain metastasis. J Clin Invest.

[135] Xing F, Liu Y, Wu SY, Wu K, Sharma S, Mo YY, Feng J, Sanders S, Jin G, Singh R (2018). Loss of XIST in Breast Cancer Activates MSN-c-Met and Reprograms Microglia via Exosomal miRNA to Promote Brain Metastasis. Cancer Res.

[136] Liu J, Ding D, Jiang Z, Du T, Liu J, Kong Z (2019). Long non-coding RNA CCAT1/miR-148a/PKCzeta prevents cell migration of prostate cancer by altering macrophage polarization. Prostate.

[137] Yu Z, Zhao H, Feng X, Li H, Qiu C, Yi X, Tang H, Zhang J (2019). Long Non-coding RNA FENDRR Acts as a miR-423-5p Sponge to Suppress the Treg-Mediated Immune Escape of Hepatocellular Carcinoma Cells. Mol Ther Nucleic Acids.

[138] Wu J, Zhang J, Shen B, Yin K, Xu J, Gao W, Zhang L (2015). Long noncoding RNA lncTCF7, induced by IL-6/STAT3 transactivation, promotes hepatocellular carcinoma aggressiveness through epithelial-mesenchymal transition. J Exp Clin Cancer Res.

[139] Zeng XY, Xie H, Yuan J, Jiang XY, Yong JH, Zeng D, Dou YY, Xiao SS (2019). M2-like tumor-associated macrophages-secreted EGF promotes epithelial ovarian cancer metastasis via activating EGFR-ERK signaling and suppressing lncRNA LIMT expression. Cancer Biol Ther.

[140] Chang L, Li J, Ding J, Lian Y, Huangfu C, Wang K (2021). Roles of long noncoding RNAs on tumor immune escape by regulating immune cells differentiation and function. Am J Cancer Res.

[141] Wu B, Yuan Y, Han X, Wang Q, Shang H, Liang X, Jing H, Cheng W (2020). Structure of LINC00511-siRNA-conjugated nanobubbles and improvement of cisplatin sensitivity on triple negative breast cancer. FASEB J.

[142] Yuan Y, Li E, Zhao J, Wu B, Na Z, Cheng W, Jing H (2021). Highly penetrating nanobubble polymer enhances LINC00511-siRNA delivery for improving the chemosensitivity of triple-negative breast cancer. Anticancer Drugs.

[143] Tai Z, Ma J, Ding J, Pan H, Chai R, Zhu C, Cui Z, Chen Z, Zhu Q (2020). Aptamer-Functionalized Dendrimer Delivery of Plasmid-Encoding lncRNA MEG3 Enhances Gene Therapy in Castration-Resistant Prostate Cancer. Int J Nanomedicine.

